# Electrochemically reduced water exerts superior reactive oxygen species scavenging activity in HT1080 cells than the equivalent level of hydrogen-dissolved water

**DOI:** 10.1371/journal.pone.0171192

**Published:** 2017-02-09

**Authors:** Takeki Hamasaki, Gakuro Harada, Noboru Nakamichi, Shigeru Kabayama, Kiichiro Teruya, Bunshi Fugetsu, Wei Gong, Ichiro Sakata, Sanetaka Shirahata

**Affiliations:** 1 Department of Bioscience and Biotechnology, Faculty of Agriculture, Kyushu University, Hakozaki, Higashi-ku, Fukuoka, Japan; 2 Nihon Trim Co. Ltd, Oyodonaka, Kita-ku, Osaka, Japan; 3 Innovation Policy Research Center, IPRC, The University of Tokyo, Hongo, Bunkyo-ku, Tokyo, Japan; 4 Policy Alternative Research Institute, The University of Tokyo, Yayoi, Bunkyo-ku, Tokyo, Japan; University of Arkansas for Medical Sciences College of Pharmacy, UNITED STATES

## Abstract

Electrochemically reduced water (ERW) is produced near a cathode during electrolysis and exhibits an alkaline pH, contains richly dissolved hydrogen, and contains a small amount of platinum nanoparticles. ERW has reactive oxygen species (ROS)-scavenging activity and recent studies demonstrated that hydrogen-dissolved water exhibits ROS-scavenging activity. Thus, the antioxidative capacity of ERW is postulated to be dependent on the presence of hydrogen levels; however, there is no report verifying the role of dissolved hydrogen in ERW. In this report, we clarify whether the responsive factor for antioxidative activity in ERW is dissolved hydrogen. The intracellular ROS scavenging activity of ERW and hydrogen-dissolved water was tested by both fluorescent stain method and immuno spin trapping assay. We confirm that ERW possessed electrolysis intensity-dependent intracellular ROS-scavenging activity, and ERW exerts significantly superior ROS-scavenging activity in HT1080 cells than the equivalent level of hydrogen-dissolved water. ERW retained its ROS-scavenging activity after removal of dissolved hydrogen, but lost its activity when autoclaved. An oxygen radical absorbance capacity assay, the 2,2-diphenyl-1-picrylhydrazyl assay and chemiluminescence assay could not detect radical-scavenging activity in both ERW and hydrogen-dissolved water. These results indicate that ERW contains electrolysis-dependent hydrogen and an additional antioxidative factor predicted to be platinum nanoparticles.

## Introduction

It has been reported that tap water purifier coverage rate reached almost 60% in an urban area of Japan [1]. The higher distribution rate of the purifiers arises from customers’ increasingly higher health-oriented desire to drink purer and safer water, which is free from tap water contaminants such as chlorine and its derivative trihalomethane, hormone-disrupting chemicals, aluminums and rust particles leaching from the inner walls of aged water pipes. Moreover, people are also demanding that these purifying apparatuses positively promote their health. Among the purifiers, alkaline ionized water generators have attracted attention because the produced water is not only tasty but also beneficial to human health. Consequently, 200,000 apparatuses have been sold each year in Japan [2]. An alkaline ionized water generator produces electrolyzed water on the surface of the cathode during electrolysis where produced water is commonly referred to as electrochemically reduced water (ERW) [3]. Typically, apparatuses that produce ERW are composed of two units, a micro-carbon cartridge unit for removal of contaminants and an electrolysis unit that acts on the purified water. Purified tap water passed through a micro-carbon cartridge unit flows into the electrolysis unit, which is composed of five platinum (Pt)-coated electrode plates, separated by semi-permeable membranes and the water is electrolyzed while passing through the gaps between the electrodes [3]. ERW exhibits suppressive effects against oxidative stress, which correlates with DNA protection [4,5], carbon tetrachloride-induced liver damage [6], lifespan extension of *Caenorhabditis elegans* [7,8], alloxan-induced type 1 diabetes [5,9], hemodialysis-induced oxidative stress during end-stage renal disease [10,11] and lipopolysaccharide-induced neuroinflammation [12].

In recent years, many studies have been conducted on the antioxidative effects of hydrogen (H_2_) gas since the report that H_2_ gas could reduce cytotoxic oxygen radicals in cultured cells and protect ischemia-reperfusion injury in rats [13]. The consumption of H_2_-enriched water has been shown to suppress the expression of pro-inflammatory cytokines by regulating gene expression [14]. Moreover, the beneficial effects of molecular H_2_ were substantiated by an increasing number of reports, including the atherosclerotic plaque stabilizing effect [15], the protective effect from peroxynitrite derived from nitric oxide [16], reductions in the number of injuries caused by hypoxia/reoxygenation [17] and cecal ligation [18]. Based on these reports, ERW and H_2_-dissolved water appear to share a common anti-oxidative effect *in vitro* and *in vivo*. ERW contains highly dissolved H_2_ (H_2_ concentration = 0.4–0.9 ppm) compared with conventional tap water (H_2_ concentration = 0 ppm), implicating strongly that its antioxidative activity is conferred by the effect of dissolved H_2_; however, there is no report that has investigated the antioxidative activity differences between ERW and H_2_-dissolved water. Therefore, the purpose of this study was to clarify the difference between ERW and H_2_-dissolved water. We report that ERW and H_2_-dissolved water exhibit distinct intracellular ROS scavenging activity in cultured cells.

## Materials and methods

### Chemicals

Dulbecco’s modified Eagle’s medium was purchased from Nissui Pharmaceutical Co., Ltd. (Tokyo, Japan). All solutions were prepared using ultrapure water from a Milli-Q® Synthesis system (Millipore, Tokyo, Japan). Individual gas cylinders each containing O_2_ (purity, >99.7%; quality, N_2_ < 1000 ppm, H_2_O < 10 ppm), N_2_ (purity, >99.999%; quality, O_2_ < 2 ppm, CO < 1 ppm, CO_2_ < 1 ppm, CH_4_ < 1 ppm, H_2_O < 5 ppm), and H_2_ (purity, >99.998%; quality, O_2_ < 1 ppm, N_2_ < 10 ppm, CO < 1 ppm, CO_2_ < 1 ppm, CH_4_ < 1 ppm, H_2_O < 5 ppm) were obtained from Fukuoka Sanso Co., Ltd. (Fukuoka, Japan). Trolox is an antioxidant and commonly used as the positive control for ROS scavenging activity measurements (Tokyo Chemical Industry Co., Ltd., Tokyo, Japan). Other reagents not detailed herein were obtained from Wako Pure Chemical Inc. (Tokyo, Japan).

### Electrochemically Reduced Water (ERW)-producing apparatus and preparation of sample water

Nihon Trim Co. Ltd. has the highest share in the market for alkaline water producers. Thus, we used a water flow-type apparatus (Trim Ion Hyper, Nihon Trim Co. Ltd., Osaka, Japan) as the test apparatus. The test apparatus has the same structure to the previously reported apparatus [3]. Briefly, the test apparatus contains two units, a micro-carbon cartridge unit and an electrolysis unit. Tap water flows into the cartridge unit and passes through the nonwoven-fabric filter, activated charcoal powders and cationic ion-exchange material to remove most of the impurities. Purified tap water then flows into the electrolysis unit, and the water is electrolyzed while passing through the gaps between the electrodes. Electrolyzed tap water near the cathode typically exhibits a high pH, a low amount of dissolved oxygen, a high negative redox potential and a high concentration of dissolved hydrogen (0.4–0.9 ppm). Water produced in this manner, with the above characteristics, is designated as ERW. The apparatus is designed to produce five types of water; four types of ERW: levels 1 (ERW_LV1_), 2 (ERW_LV2_), 3 (ERW_LV3_) and 4 (ERW_LV4_) electrolyzed with a constant electric current for each level (0.8 to 4.2 A) at a maximum of 50 volts while passing through the gaps between the electrodes equipped in the test apparatus and one type of filtered water without electrolysis (FW). We also prepared two types of water as controls: MilliQ (MQ) water and filtered water, both are saturated with mixed gas. The mixed gas consisted of 80% H_2_, 15% O_2_, and 5% N_2_, and this gas was bubbled through the water to achieve the same dissolved oxygen (DO) and dissolved H_2_ (DH) concentration to those of ERW_LV4_. The pH was adjusted to match ERW_LV4_ using sodium hydrate.

### Measurement of pH, dissolved gas, EC and ORP of sample water

Dissolved H_2_ concentrations were measured using a DM-10B2 meter (ABLE Corporation, Tokyo, Japan). Degassed ultrapure water was saturated with H_2_ gas by bubbling for 30 min. This solution was defined as standard H_2_-saturated (H_2_Sat) water. Dissolved H_2_ concentrations in sample solutions were calculated using H_2_Sat as the standard value.

Dissolved O_2_ was measured using an YSI-3000 dissolved O_2_ meter (YSI/Nanotech Inc. Kanagawa, Japan). The oxygen concentration was determined by setting an air-saturated MQ water, which contains 7.87 ppm of oxygen at 1 atm and 27°C, as 100% [19]. We also measured the oxidation-reduction potential (ORP) (F-52 with electrode, 9300-10D, HORIBA, Ltd., Tokyo, Japan), electrical conductivity (EC) (CM-14P, DDK-TOA, Co., Tokyo, Japan) and pH (F-52 with electrode, 9618, HORIBA, Ltd., Tokyo, Japan).

### Oxygen Radical Absorbance Capacity (ORAC) assay and 2,2-diphenyl-1-picrylhydrazyl (DPPH) assay

The ORAC assay measures the change of probe fluorescence in response to the scavenging of peroxyl radicals generated by 2,2ʹ-azobis (2-methylpropionamidine) dihydrochloride (AAPH) by antioxidants that prevent the degradation of the fluorescein probe. The analysis of the ORAC assay was carried out following the method by Huang et al. [20]. Briefly, 20 μL of 15 μM fluorescein dissolved in a 100 mM phosphate buffer (pH 7.0) solution was added to the designated wells of a 96-well black plate, followed by the addition of 25 μL of blank, standard (Trolox (6-hydroxy-2,5,7,8-tetramethylchroman-2-carboxylic acid) 5–40 μM), or sample to the wells. Then, 15 μL of freshly prepared 30 mM 2,2′-azobis (2-methylpropionamidine) dihydrochloride solution was added to all wells. Fluorescence was monitored using 485 nm of excitation and 528 nm of emission at 10-min intervals for 180 min to generate fluorescence intensity versus time plots for samples, blank and Trolox. Using the plots, the net area under the curve for the sample was calculated by subtracting the integrated values of the area under the curve for the blank from that of the sample and Trolox. The value for individual samples was then obtained by subtracting the net Trolox value from that of the samples.

An adopted method of the 2,2-diphenyl-1-picrylhydrazyl (DPPH) assay by Brand-Williams et al. was used for measuring total antioxidative capacity [21]. The method measures the absorbency reduction of stable DPPH nitrogen radicals by the action of antioxidants. Trolox is commonly used to determine the equivalent value of an antioxidant [22,23]. Briefly, 500 μM of DPPH was prepared in ethanol or acetonitrile as the stock solution. Reaction cocktails consisted of 50 μL of either blank, Trolox (positive control) or samples, 10 μL of acetate butter (pH 5.5) and 40 μL of DPPH were mixed, and kept in the dark for 30 min. The absorbance values were measured at 515 nm using a microplate reader. A standard curve was prepared using 50–250 μM Trolox.

### Cell culture, measurement of cell viability and intracellular ROS

HT1080 cells (CCL-121; American Type Culture Collection, Manassas, VA, USA), a human fibrosarcoma cell line, were cultured with medium supplemented with 10% (v/v) fetal bovine serum (Lot No. 12D168, Sigma-Aldrich Japan Co. LLC, Tokyo, Japan). The cell number was measured using a cell counter (Blood cell counting device F-520, Sysmex Co., Hyogo, Japan). HT1080 cells at a density of 0.5 × 10^5^ cells in 100 μL DMEM were used to inoculate wells of 96-well plates and cultured for 24 h in a CO_2_ gas incubator. Then, the cells were placed in a medium containing various sample waters with or without hydrogen peroxide (H_2_O_2_). Briefly, cells were first treated with 60 μM H_2_O_2_ for 30 min and replaced with a medium made from 14 types of water, followed by 120 min of incubation. Sample media were prepared immediately before use. This was done by mixing 5× concentrated DMEM medium with sample water at a ratio of 1:4. Cells were then incubated with 2 μM 3′-O-acetyl-6′-O-pentafluorobenzenesulfonyl-2′,7′-difluorofluorescein (BES-H_2_O_2_-AC) [24] and 1 μM Hoechst 33342 for 15 min. The intracellular fluorescence intensity generated by the sample waters was probed with BES-H_2_O_2_-AC designed to detect specifically intracellular H_2_O_2_ and the fluorescence levels were detected using an IN Cell Analyzer 1000 (GE Healthcare, Tokyo, Japan) with an excitation filter at 480 nm and an emission filter at 535 nm. Data derived from 1350 cells were statistically analyzed. The IN Cell Analyzer 1000 was able to rapidly and quantitatively determine the fluorescence intensity of BES-H_2_O_2_-AC in each well to provide statistically reliable data derived from several thousand to several million cells. In this study, we used a specific lot of fetal bovine serum that we empirically determined as the best-suited serum that ensured high sensitivity and reproducibility for the detection of intracellular ROS scavenging activity in HT1080 cells. We provide more detail in the Supporting Information. We also performed immuno spin trapping assay to evaluate the antioxidative activity of ERW. Immuno spin trapping assays with 5,5-dimethyl-1-pyrroline N-oxide (DMPO; Labotec Co., Tokyo, Japan) were performed by one of the following three procedures: 1. Cells were treated with 80 μM H_2_O_2_ for 30 min, which was replaced with water sample medium with DMPO for 30 min. 2. Cells were treated with 80 μM H_2_O_2_, water sample medium and DMPO at the same time for 90 min. 3. Cells were treated with 80 μM H_2_O_2_ or 100 μM menadione for 30 min, which was replaced with water sample medium followed by 90 min of incubation. In all of these three alternatives, cells were then incubated with 40 mM DMPO for 30 min. Sample media were prepared immediately before use. This was done by mixing 5× concentrated DMEM medium with sample water at a ratio of 1:4. Then, cells were fixed with 4% paraformaldehyde/phosphate-buffered saline (PBS) on ice. Anti-DMPO nitrone adduct antibody (ab110430; Abcam, Cambridge, UK) was used at 1:1000 dilution with PBS-T/1% bovine serum albumin after treatment with PBS/0.25% Triton X for 10 min. After that, cells were incubated with fluorescein-labeled avidin (1:400 dilution; Fluorescein Avidin DCS, cell sorting grade; Vector Laboratories Ltd., Burlingame, CA, USA) and 1 μM Hoechst 33342 for 60 min. The intracellular fluorescence intensity was detected using an IN Cell Analyzer 1000 (GE Healthcare, Tokyo, Japan) with an excitation filter at 480 nm and an emission filter at 535 nm. The IN Cell Analyzer 1000 can rapidly and quantitatively determine the fluorescence intensity in each well to provide statistically reliable data derived from several thousand to several million cells. We established this protocol to detect weak intracellular oxidation. Therefore, hydrogen peroxide treatment is used to induce oxidation and/or injury to the intracellular components of cells. The probe is expected to detect the production of intracellular ROS resulting from such injury of organelles, from oxidized cellular molecules, from the process of recovery of oxidized molecules, and from the retention of intracellular hydrogen peroxide. There are additional information for redetection of intracellular scavenging activity of ERW (S1 Supporting Information)

### ICP-MS analysis of ERW

Ten milliliters of water samples (MQ, FW, LV1, LV2, LV3, LV4) were cleaned by filtration and analyzed semi-quantitatively for elements present in the samples using ICP-MS (Agilent 7500c, Agilent Technologies Co. Ltd., Santa Clara, CA, USA) at the Center of Advanced Instrumental Analysis of Kyushu University.

### Observation of platinum electrode by Scanning Electron Microscopy (SEM)

The structure of the surface and cross-section of the electrode plate were observed under a field-emission scanning electron microscope (JEOL-6390, JEOL Ltd. Tokyo, Japan). Photographs were taken at 10 kV with magnifications of 10,000 or 30,000.

### Statistical analysis

Microsoft Excel 2016 was used to calculate the means, standard deviations or coefficient of determinations in triplicate experiments. One- or Two-way ANOVA (STATMATE III for Windows) was used to determine if there were statistically significant differences.

## Results and discussion

### Characterization of several types of water

Parameters such as pH, EC, ORP, DO and DH were measured for several types of water, including MQ, tap water, FW and ERWs (ERW_LV1–LV4_). FW is filtered water by passing tap water through the micro-carbon cartridge without electrolysis. ERW_LV1_ is ERW generated by electrolyzing filtered water at level 1 with constant electric current at 50 V upper limit voltage and a flow rate of 1.8–2.0 l/min. Likewise, other ERWs were produced using identical conditions, except selection of the LV2, LV3 or LV4 switch. As shown in Table 1, pH, ORP negativities and DH values increased and were dependent on the electrolysis strength, whereas EC and DO remained essentially constant. Such fundamental characteristics of ERWs produced by the test apparatus are in agreement with previously published data, demonstrating the reproducibility of the apparatus [3,9]. Using these waters as starting materials, we examined whether the ERWs have ROS scavenging activity in HT1080 cells after H_2_O_2_ stimulation.

**Table 1:** Characteristics of water samples.

	pH	EC (mS/m)	ORP (mV)	DO (ppm)	DH (ppm)
**MQ**	N.D.	0 ± 0.1	191 ± 10	7.9 ± 0.9	N.D.
**FW**	8.3 ± 0.1	38.2 ± 2.3	127.1 ± 25.1	7.4 ± 0.2	N.D.
**ERW**_**LV1**_	9.4 ± 0.1	38.5 ± 0.1	−90.7 ± 4.5	7.7 ± 0.7	0.2 ± 0.2
**ERW**_**LV2**_	9.3 ± 0.1	38.7 ± 0.3	−99.2 ± 1.4	7.8 ± 0.2	0.3 ± 0
**ERW**_**LV3**_	9.5 ± 0.1	38 ± 0.1	−164 ± 36.7	7.1 ± 0.4	0.5 ± 0.1
**ERW**_**LV4**_	10 ± 0.2	38.2 ± 2.3	−675.1 ± 49.8	5.8 ± 0.5	0.9 ± 0.2

MQ, MilliQ water; FW, filtered water by passing tap water through the nonwoven-fabric filter, activated charcoal powders and cationic ion-exchange material. ERW_LV1-LV4_, ERW generated by electrolyzing filtered water at level 1–4 with a constant electric current of 50 V (upper limit) and a flow rate of 1.0–1.2 l/min.

**Fig 1:**
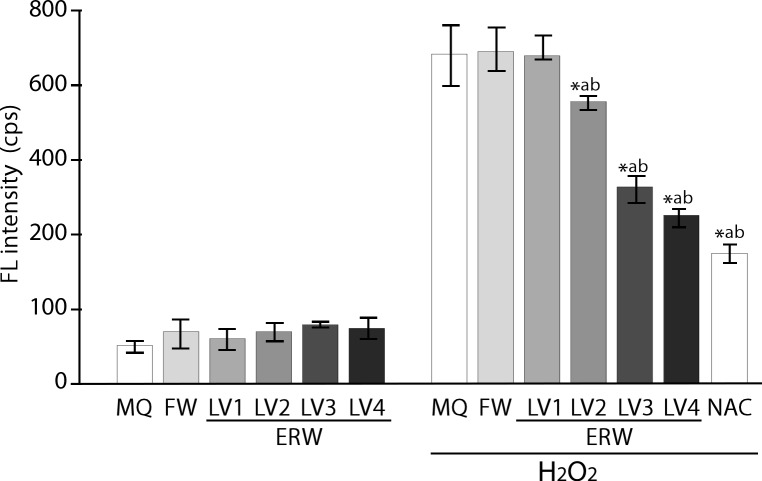
Detection of the intracellular ROS-scavenging activity of ERW using BES-H_2_O_2_ probe. MQ, MilliQ water; FW, filtered water by passing tap water through the nonwoven-fabric filter, activated charcoal powders and cationic ion-exchange material. Detailed information is given in ref. [3] LV1, LV2, LV3 and LV4 represent FW electrolyzed at levels 1, 2, 3 and 4 with a maximum of 50 V using the test apparatus. NAC, 5 mM N-acetyl cysteine. *a and *b indicate *p* values of < 0.01 when compared with the value for H_2_O_2_-treated MQ and FW, respectively.

As shown in Fig 1, the Bes-H_2_O_2_-AC probe detected basal intracellular fluorescence (FL) in HT1080 cells treated with all sample waters, including MQ, FW and ERW_LV1_‒ERW_LV4_ (Fig 1, left). The FL intensity was found to increase only when treated with 60 μM of hydrogen peroxide for 30 min (Fig 1, right). ERW_LV1_-treated cells did not show a difference in this regard from those treated with FW; however, intracellular FL intensities in the cells treated with ERW_LV2_‒ERW_LV4_ were found to decrease significantly and in an electrolysis intensity-dependent manner (Fig 1, right). The sample waters have different pH, as shown in Table 1; however, we confirmed that the pH levels of all of the prepared media containing the sample waters had been adjusted using medium buffer to the range of 7.39 to 7.49 (Sl Table). We used 5 mM of *N*-acetyl cysteine (NAC) as a positive control, which showed a significant reduction of H_2_O_2_ induced intracellular ROS levels. Thus, confirming that the assay system was working properly. NAC was used here because it acts as a direct ROS scavenger for hydrogen peroxide, the superoxide radical and hydroxyl radical, and as an indirect ROS scavenger by functioning as a precursor of reduced glutathione. Therefore, electrolysis levels are closely correlated with dissolved hydrogen levels and ROS scavenging ability. To deepen our understanding, we prepared two types of water as controls: (i) MQ water and (ii) FW, in which both are saturated with mixed gas (MQ+Mixed gas, FW+Mixed gas, parameters shown in Table 2)

**Table 2:** Characteristics of other water samples.

Sample water	Treatment	pH	EC (mS/m)	ORP (mV)	DO (ppm)	DH (ppm)
**MQ**	None	N.D.	0.1 ± 0.03	243.7 ± 40.6	7.7 ± 0.2	N.D.
**MQ**	+Mixed gas	10.1 ± 0.1	0.1 ± 0.04	−334 ± 42.8	5.3 ± 0.1	0.8 ± 0.1
**MQ**	+Mixed gas +degas	10.0 ± 0.1	0.2 ± 0.02	309.7 ± 27.5	7.4 ± 0.3	N.D.
**MQ**	+H_2_Sat	N.D.	0.1 ± 0.11	−389.8 ± 50.2	3.1 ± 0.2	1.6 ± 0.2
**Tap w**	None	8.3 ± 0.2	38.3 ± 2.1	431.7 ± 41.3	7.8 ± 0.5	N.D.
**FW**	None	8.1 ± 0	40.2 ± 1.7	282.2 ± 2.4	7.8 ± 0.1	N.D.
**FW**	+Mixed gas	10.0 ± 0.1	41.8 ± 1.6	−344.8 ± 35.6	5.1 ± 0.4	0.9 ± 0.2
**FW**	+Mixed gas +degas	9.8 ± 0.1	44.6 ± 0.5	256.7 ± 18.5	7.5 ± 0.1	N.D.
**FW**	+Mixed gas +autoclave	8.0 ± 0.3	24.7 ± 1.5	152.3 ± 28.6	7.3 ± 0.2	N.D.
**ERW**_**LV4**_	None	10.5 ± 0.1	24.7 ± 0.8	−602.2 ± 48	5.4 ± 0.6	0.9 ± 0.2
**ERW**_**LV4**_	+degas	10.0 ± 0.5	23.7 ± 1.0	111.3 ± 21.9	7.2 ± 0.3	N.D.
**ERW**_**LV4**_	+autoclave	9.7 ± 0.1	14.8 ± 0.8	99.1 ± 38.7	7.3 ± 0.1	N.D.

MQ, MilliQ water; Tap w, Tap water; FW, filtered pure water; ERW_LV4_, ERW at electrolysis level 4. +Mixed gas, H_2_, O_2_ and N_2_ mixed gas was bubbled through water to obtain DO and DH concentrations that are equal to those of ERW_LV4_. The pH was also adjusted using sodium hydrate. +H_2_Sat, H_2_ saturated water; +degas, Degassed; +autoclave, autoclaved. N.D.: Not detected.

**Fig 2:**
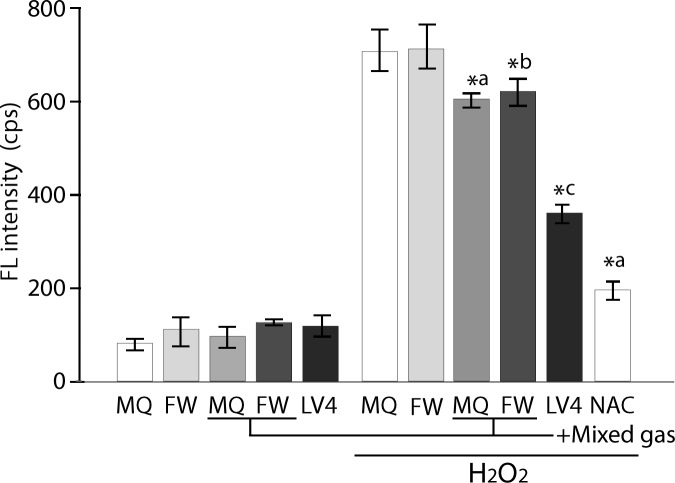
Measurement of the intracellular ROS intensity of controls and ERW using BES-H_2_O_2_ probe. MQ, MilliQ water; FW, filtered pure water; +Mixed gas, H_2_, O_2_ and N_2_ mixed gas was bubbled through water to obtain DO and DH concentrations that are equal to those of LV4. The pH was also adjusted using sodium hydrate.; LV4, ERW at electrolysis level 4. NAC, 5 mM N-acetyl cysteine. *a, *b and *c indicate *p* values of < 0.01 when compared with the value for H_2_O_2_-treated MQ, FW and +Mix gas of FW, respectively.

The control waters and plain MQ, FW and ERW_LV4_ were assessed for their intracellular ROS scavenging activity. All five types of water showed basal FL levels (Fig 2, left). In contrast, the cells cultured with plain MQ and FW-based medium treated with 60 μM H_2_O_2_ for 30 min showed approximately 750 cps of FL intensity, referring to them as absolute controls, whereas these two types of water saturated with mixed gas significantly reduced the intracellular FL intensity when compared with that of the absolute controls (Fig 2, right). Moreover, the cells cultured with ERW_LV4_ based medium significantly reduced the intracellular FL intensity when compared with that of the absolute controls and mixed gas saturated waters. NAC (5 mM, 30 min) as a positive control exerted an expected antioxidative effect (Fig 2, right).

**Fig 3:**
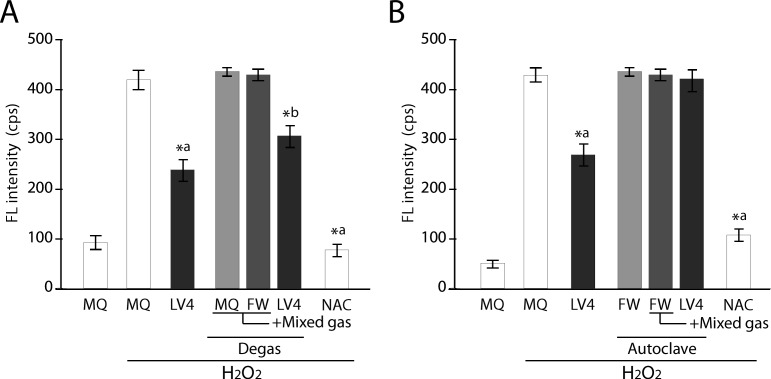
Measurement of intracellular ROS intensity of degassed and autoclaved controls and ERW using BES-H_2_O_2_ probe. **A) Comparison of degassed sample water vs. ERW. B) Comparison of autoclaved sample water vs. ERW.** MQ, MilliQ water; FW, filtered pure water; +Mixed gas, H_2_, O_2_ and N_2_ mixed gas was bubbled through water to obtain DO and DH concentrations that are equal to those of LV4. The pH was also adjusted using sodium hydrate. LV4, ERW at electrolysis level 4. NAC, 5 mM *N*-acetyl cysteine. Degas, Degassed samples. Autoclave, autoclaved samples. *a and *b indicate *p* values of < 0.01 when compared with the value for H_2_O_2_-treated MQ and degassed +Mix gas of FW, respectively.

To confirm whether intracellular ROS scavenging activity is conferred by H_2_ gas, we treated the water with degassing and autoclaving steps, because such treatments are thought to eliminate and purge gases, respectively [5,25]. Intracellular ROS scavenging activity in the cells treated with the degassed ERW_LV4_-based medium still showed significant ROS reduction; although the level was slightly weaker than that of the sample without degas treatment (Fig 3A). This is important because the ROS scavenging activity remains in ERW_LV4_ after degassing treatment, thus indicating the existence of non-gaseous ROS scavenging factors in addition to gaseous factors (e.g., H_2_). We then examined the effect of an autoclaving step for ROS scavenging ability. The result demonstrated that the cells cultured with the autoclaved ERW_LV4_-based medium completely lost intracellular ROS scavenging activity, whereas a consistent ROS scavenging effect was obtained with non-autoclaved ERW_LV4_-based medium (Fig 3B). These results suggest that the prospective antioxidative factor has heat and pressure labile characteristics.

To elucidate the nature of the prospective antioxidative factors contained in ERW_LV4_, we first prepared several types of water simulating the ERW_LV4_ characteristics (Table 2) by adjusting the pH, DO and DH of MQ and FW by bubbling the mixed gas with N_2_, O_2_ and H_2_. These sample water-based culture media were used to culture HT1080 cells after either degassing or autoclave treatment, and the intracellular ROS scavenging activity was measured using BES-H_2_O_2_-AC after the addition of H_2_O_2_. Degassed and H_2_O_2_-stimulated sample waters (MQ, FW) could not reduce intracellular ROS, but ERW_LV4_ and NAC reduced ROS significantly (Fig 3A). In contrast, the same experiments using the autoclaved waters showed that ERW_LV4_, but not NAC, lost its ROS scavenging activity (Fig 3B). Therefore, the results indicate that the antioxidative factors in ERW_LV4_ are produced during electrolysis.

**Fig 4:**
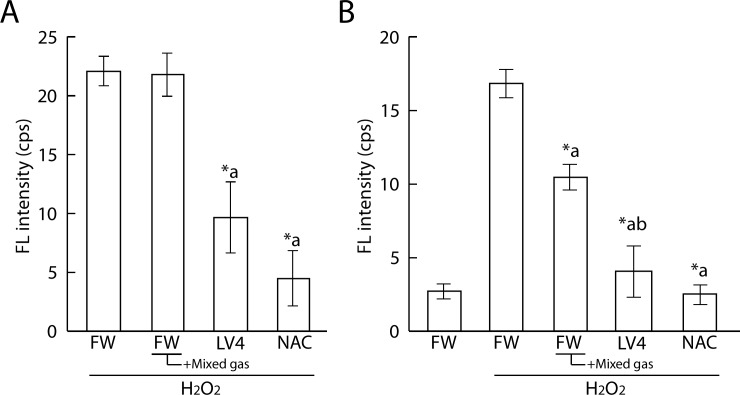
Measurement of protein radicals of ERW and dissolved H_2_ water by immuno spin trapping assay. A. 60 mM DMPO and sample water were applied for 90 min after treatment with 80 μM H_2_O_2_ for 30 min. B. 40 mM DMPO, sample water and 80 μM H_2_O_2_ were applied at the same time for 90 min. FW, filtered pure water; +Mixed gas, H_2_, O_2_ and N_2_ mixed gas were bubbled through water to obtain DO and DH concentrations equal to those of LV4 –the pH was also adjusted using sodium hydrate; LV4, ERW at electrolysis level 4. NAC, 5 mM N-acetyl cysteine. *a, *b indicate p values of < 0.01 when compared with the values for H_2_O_2_-treated FW and +Mixed gas FW, respectively.

We also performed immuno spin trapping assays of ERWs to confirm the antioxidative activity of ERW. We firstly tested effects of treatment with the identical BES-H_2_O_2_-AC protocol but could not detect a difference of FL intensity in cells treated with H_2_O_2_ at concentrations of between 0 and 60 μM (data not shown). Then, we changed the treatment order as follows: Cells were treated with 80 μM H_2_O_2_ for 30 min, which was then replaced with water sample medium with DMPO for 30 min (Fig 4A). As a result, we detected a significant decrease in FL intensity in LV4 and NAC sample water, but not in mixed gas FW. Therefore, we next changed the treatment order as follows: treatment with 40 mM DMPO, sample water and 80 μM H_2_O_2_ at the same time for 90 min (Fig 4B). As a result, we detected significant differences between FW and FW+mixed gas, FW and LV4, and FW+mixed gas and LV4. These results suggest that DMPO requires a certain volume of hydrogen peroxide and a supply of oxygen radicals nearby to produce DMPO adducts. Therefore, to establish similar conditions in our study using a BES-H_2_O_2_ probe, we additionally performed experiments using menadione instead of hydrogen peroxide.

**Fig 5:**
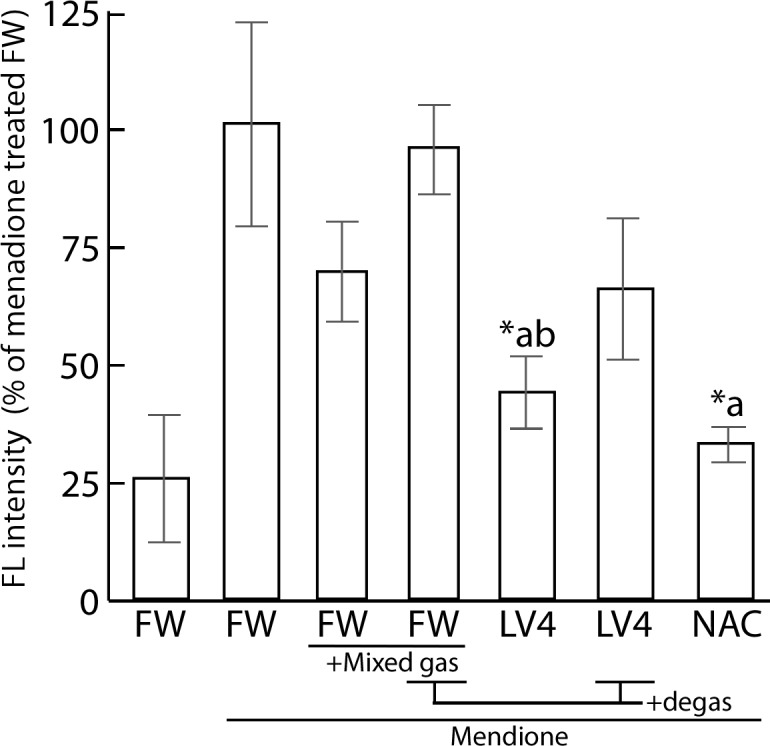
Measurement of protein radicals of ERW and dissolved H_2_ water by immuno spin trapping assay. Cells were initially treated with 100 μM menadione for 45 min, which was replaced with water media for 120 min. Then, 60 mM DMPO was applied for 30 min. FW, filtered pure water; +Mixed gas, H_2_, O_2_ and N_2_ mixed gas were bubbled through water to obtain DO and DH concentrations equal to those of LV4 –the pH was also adjusted using sodium hydrate; LV4, ERW at electrolysis level 4. NAC, 5 mM N-acetyl cysteine. Degas, Degassed samples. *a and *b indicate p values of < 0.01 when compared with the values for menadione-treated FW and menadione-treated +Mixed gas FW, respectively.

As shown in Fig 5, the FL intensity was increased upon treatment with 80 μM menadione for 45 min. The CL intensity of cells which cultured with LV4-contained medium significantly decreased and FW+Mixed gas had a tendency to reduce the intracellular FL intensity when compared with that of the absolute controls. NAC was used as a positive control. In addition, LV4 and FW seemed to lose ROS scavenging activity upon degas treatment; moreover, LV4 degassed water showed a tendency for persistence in its activity.

**Fig 6:**
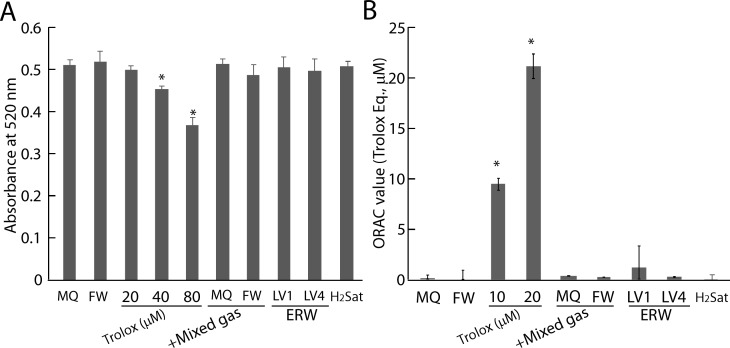
Result of DPPH assay and ORAC assay of H_2_-dissolved water and ERW. A. DPPH assay of sample water. B. ORAC assay of sample water. MQ, MilliQ water; FW, filtered pure water; +Mixed gas, H_2_, O_2_ and N_2_ mixed gas was bubbled to obtain DO and DH concentrations equal to those of LV4. The pH was adjusted using sodium hydrate. LV1 and LV4, ERW at electrolysis levels of 1 and 4, respectively. H_2_Sat, hydrogen saturated water. * indicates *p* values of < 0.01 when compared with the value for MQ.

In parallel with this experiment, sample waters were tested to determine whether the prospective antioxidative factors exert direct ROS scavenging activity for the DPPH radical and hydrogen atom transfer-based ORAC assays. Trolox was used as a positive control as well as a system control, and was confirmed to reduce absorbency in a concentration-dependent manner where 40 μM and 80 μM Trolox significantly reduced the absorbency at 520 nm compared with MQ in the DPPH assay (Fig 6A). However, FW, H_2_-dissolved water (plus the gas mix) of MQ and FW and H_2_-saturated water (H_2_Sat) as well as ERW_LV1_ and ERW_LV4_ did not show DPPH radical scavenging activity (Fig 6A). Similarly, 10 μM of Trolox showed ROS scavenging activity, whereas FW, +H_2_ gas of MQ and FW, H_2_Sat, ERW_LV1_ and ERW_LV4_ did not show 2,2′-Azobis 2-amidinopropane dihydrochloride-derived ROS-scavenging activity in the ORAC assay (Fig 6B). Thus, the DPPH and ORAC assays indicated that the prospective antioxidative factors did not involve stable radical reduction and hydrogen-atom-donation reactions. However, complete exclusion of weak antioxidative activities by the factors cannot be ruled out completely, because the detection limit of DPPH and ORAC is ~10 μM and 1 μM Trolox equivalent, respectively. We additionally measured the ROS-scavenging activity of dissolved hydrogen using a chemiluminescence assay because this assay has a more sensitive ROS response, which can be seen in the supporting information (S2 Supporting Information and S1 Fig).

**Fig 7:**
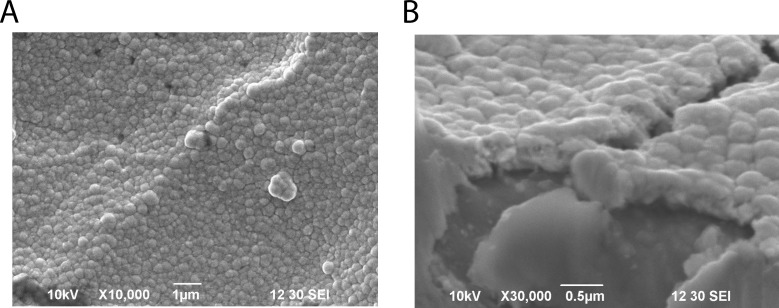
Observation of the electrode plate surface by SEM. A. Image of the electrode plate surface. Bars represent 1 μm. B. Cross-section of the electrode plate. Bars represent 0.5 μm.

Based on the present results, the prospective antioxidative factors are considered to be dissolved H_2_ and non-gaseous materials with heat and pressure lability; both species are produced in an electrolysis strength-dependent fashion. To further specify the latter prospective factors, we analyzed elements contained in various types of water using semi-quantitative ICP-MS analysis (S2–S4 Figs). We searched for an element from 62 elements that increased in amount in an electrolysis-dependent manner, yet existed at low levels in the absence of electrolysis. Most elements were found to show minimal change and exist at low levels. However, Pt was found to increase in an electrolysis-dependent manner. This tentative conclusion is probable because we reported previously that ERW contains platinum nanoparticles (Pt NPs), as visualized by transmission electron microscopy [26]. It has been suggested that the elevated Pt NPs in ERW originate from the platinum electrode equipped in the ERW producing apparatus [26]. Further support for the presence of Pt NPs comes from a report that Pt NPs form aggregates upon autoclave treatment and such aggregates no longer show ROS scavenging activity [27]. Our data and previous reports suggested Pt NPs exist in ERW, but the process of formation of Pt NPs has not been considered in depth. It has been reported that ERW could contain metal and/or nonmetal nanoparticles eluted from electrode ions during electrolysis only when electrodes are constructed with a metal with a high ionization tendency, which excludes platinum as a candidate metal [28]. However, an alternative view suggests that the surface structure of metal-coated plates could be formed as a layer of very tiny globular structure [29,30]. The presently used apparatus contains platinum-coated plate electrodes and the surface structure appears to be a sheet of aggregated nanoparticles (Fig 7). Although SEM magnification could not clearly reveal nanometer-sized globular structures, it is assumed that they are nanometer-sized particle-like structures, as suggested previously [29,30]. These reports lead us to consider that electrolysis increases the number of Pt NPs in ERW due to physical force-detachment during electrolysis, and the produced Pt NPs are a potential active factor of the ROS-scavenging activity of ERW. However, a drawback to this hypothesis is that the amount of Pt NPs present in ERW is very low, with values between 0 and 0.7 ppb [7]. The Pt NPs concentration of ERW produced by the currently used apparatus is 0.2 ppb, as determined by quantitative analysis of ICP-MS data (data not shown). The molar concentration is calculated to be 20 pM based on our previous study [27]. Therefore, such a Pt NPs concentration appears to be too low to exert direct intracellular ROS scavenging activity, as observed in Fig 2. In an attempt to link the intracellular ROS scavenging activity and the trace amount of Pt NPs in ERW, we propose an indirect ROS-scavenging activity mechanism, in which the Pt NPs work as a ligand for activation of the intracellular antioxidant systems. This mechanism holds true only when an additional assumption is fulfilled; the existence of high affinity nanoparticle-receptors that have affinity comparable to low molecular weight hormone receptors present in cells [31].

## Conclusions

We verified significant electrolysis intensity-dependent intracellular ROS-scavenging activity of ERW. DPPH and ORAC assays revealed that ERW did not show radical-scavenging activity. ERW had significant reduction of intracellular ROS activity in comparison with H_2_-dissolved water. Additionally, ERW retained its ROS-scavenging activity even after removal of dissolved H_2_, but lost its activity when autoclaved. The combined results suggest that ERW contains not only dissolved H_2_ but also a trace amount of Pt NPs. These Pt NPs may act as a ligand for the activation of intracellular antioxidant systems via hypothetical receptors, which gives rise to an indirect ROS scavenging mechanism in cells.

## Supporting information

S1 Supporting information Additional information for detection of the intracellular scavenging activity of ERW and hydrogen-dissolved water. (DOCX)

S2 Supporting Information Information for measurement of superoxide anion radical scavenging activity of dissolved H_2_ water using chemiluminescence (CL) assay. (DOCX)

S1 Fig Measurement of superoxide anion radical scavenging activity of Trolox and sample water by CL assay. A. Plots of CL intensity versus log concentration of Trolox. We used N_2_ dissolved water for measurement superoxide anion radical scavenging activity of Trolox. B. Superoxide anion radical scavenging activity of Trolox and sample water. H_2_, He and N_2_ represent H_2_ dissolved water, He dissolved water and N_2_ dissolved water, respectively. *a, *b and *c indicate *p* values of < 0.01 when compared with the value for N_2_ dissolved water, 0.1 μM Trolox-containing and 0.25 μM Trolox-containing N_2_ dissolved water, respectively.
(TIF)

S2 Fig Semi-quantitative ICP-MS analysis of ERW. MQ, MilliQ water; FW, filtered water; LV1, LV2, LV3 and LV4, filtered water electrolyzed at levels 1, 2, 3 and 4 with a maximum of 50 V while passing through the gaps between the electrodes; SD, standard solution that contains 10 ppb each of lithium, yttrium and thallium in MilliQ water.
(TIF)

S3 Fig Semi-quantitative ICP-MS analysis of ERW 2. Notations are same as S2 Fig.
(TIF)

S4 Fig Semi-quantitative ICP-MS analysis of ERW 3. Notations are same as S2 Fig.
(TIF)

S1 Table pH of sample water-containing media. ERW_LV1-LV4_, ERW generated by electrolyzing filtered water at level 1–4 with a constant electric current of 50 V (upper limit) and a flow rate of 1.0–1.2 l/min.
(DOCX)
