# Supplementary material for: Electrochemically reduced water exerts superior reactive oxygen species scavenging activity in HT1080 cells than the equivalent level of hydrogen-dissolved water
Source: PLoS One. 2017 Feb 9;12(2):e0171192. doi: 10.1371/journal.pone.0171192 (PMC5300231; doi:10.1371/journal.pone.0171192)
Supplement: S1 Supporting information — (DOCX) [file pone.0171192.s001.docx]

**Additional Information for Detection of the Intracellular Scavenging Activity of ERW and Hydrogen-Dissolved Water.**

Electrochemically reduced water (ERW) produced by flow-type apparatuses using tap water as a continuous source have attracted researchers’ attention due to the intracellular ROS scavenging activity of ERW in cultured cells [1–4]. However, these studies are incomplete, in part because the antioxidative activity of ERW is rather weak and thus difficult to analyze in detail. It has been shown that serum often affects the results of cell culture experiments. With this observation, we have screened 20 lots of bovine serum that were available to us during our experimental period and selected the serum that offer results with high sensitivity and reproducibility. Therefore, serum screening should be performed prior to the intracellular ROS scavenging assays (*e.g.*, BES-H_2_O_2_ assay system) when the test samples (*e.g.*, ERWs) are expected to have lower intracellular ROS scavenging activity.
