# Supplementary material for: Electrochemically reduced water exerts superior reactive oxygen species scavenging activity in HT1080 cells than the equivalent level of hydrogen-dissolved water
Source: PLoS One. 2017 Feb 9;12(2):e0171192. doi: 10.1371/journal.pone.0171192 (PMC5300231; doi:10.1371/journal.pone.0171192)
Supplement: S2 Supporting Information — (DOCX) [file pone.0171192.s002.docx]

**Measurement of superoxide anion radical scavenging activity of dissolved H_2_ water using chemiluminescence (CL) assay.**

We additionally measured ROS-scavenging activity of dissolved hydrogen using a chemiluminescence assay because a more sensitive ROS response can be obtained through this assay. In this experiment, we selected superoxide anions radical as the radical species.

**Methods**

The reaction mixture (2 ml) for measuring CL intensity contained 100 μM hypoxanthine (Tokyo Chemical Industry Co., Ltd., Tokyo, Japan), 80 mM sodium phosphate buffer (pH 7.4), 0.9 ml of sample, 10 μM 2-Methyl-6-phenyl-3,7-dihydroimidazo[1,2-a]pyrazin-3-one (CLA, Tokyo Chemical Industry Co., Ltd.) and 1 mU/ml xanthine oxidase (XOD; Roche Diagnostics, Tokyo, Japan). The reaction mixture (1.998 ml) without XOD solution was mixed and then added to a 2-ml plastic Petri dish. Then, 2 μl of XOD solution was added and immediately mixed using a pipette. After 20 seconds, the CL intensity was measured for 30 seconds using a CLD-110 Chemiluminescence Detector (Tohoku Electronic Industrial Co. Ltd., Tohoku, Japan). The average CL intensity (cps) in each sample water was adopted to evaluate superoxide anion radical scavenging activity. The used dissolved H_2_ water is MQ+Mix gas shown in Table 2. We also prepared the following two other types of dissolved mixed gas MQ water as controls: dissolved nitrogen (N_2_) water and dissolved helium (He) water. Each mixed gas consisted of 85% H_2_ and 15% O_2_, and 80% He, 15% O_2_ and 5% N_2_, respectively. Mixed gas was bubbled through the water to achieve the same dissolved oxygen and dissolved H_2_ concentrations as those of dissolved H_2_ water. The purity and quality of helium gas (Fukuoka Sanso Co., Ltd., Fukuoka, Japan) were as follows: purity, >99.999%; quality, O_2_ < 1 ppm, N_2_ < 2 ppm, CO < 1 ppm, CO_2_ < 1 ppm, CH_4_ < 1 ppm, H_2_O < 5 ppm and CO_2_ (purity, >99.9%).

**Results**

We first measured the ROS scavenging activity of Trolox in this CL system. Under our conditions, Trolox decreased CL intensity in a dose-dependent manner with a sigmoid curve (S1A Fig). CLA excitation and emission is known to be induced depending on the oxygen free radicals generated by the hypoxanthine-xanthine oxidase (HPX-XOD) reaction system at a constant rate. From this sigmoid curve, we can speculate that CLA and Trolox shows competitive reactions against superoxide anion radical [27]. Next, we subjected the water samples to test their ROS-scavenging activity. As shown in S1B Fig, the average CL intensity of MQ water was ca. 22,000 cps under these conditions. The CL intensity of H_2_ dissolved water decreased compared with that of MQ water. However, there was no significant difference between He dissolved water and N_2_ dissolved water, while we confirmed that there were significant differences among 0.1, 0.25 and 0.5 μM Trolox solutions. These results suggest that He dissolved water, N_2_ dissolved water and H_2_ dissolved water have less than 0.1 µM Trolox equivalent superoxide anion radical scavenging activity.
