# Supplementary figures and images for: Electrochemically reduced water exerts superior reactive oxygen species scavenging activity in HT1080 cells than the equivalent level of hydrogen-dissolved water
Source: PLoS One. 2017 Feb 9;12(2):e0171192. doi: 10.1371/journal.pone.0171192 (PMC5300231; doi:10.1371/journal.pone.0171192)

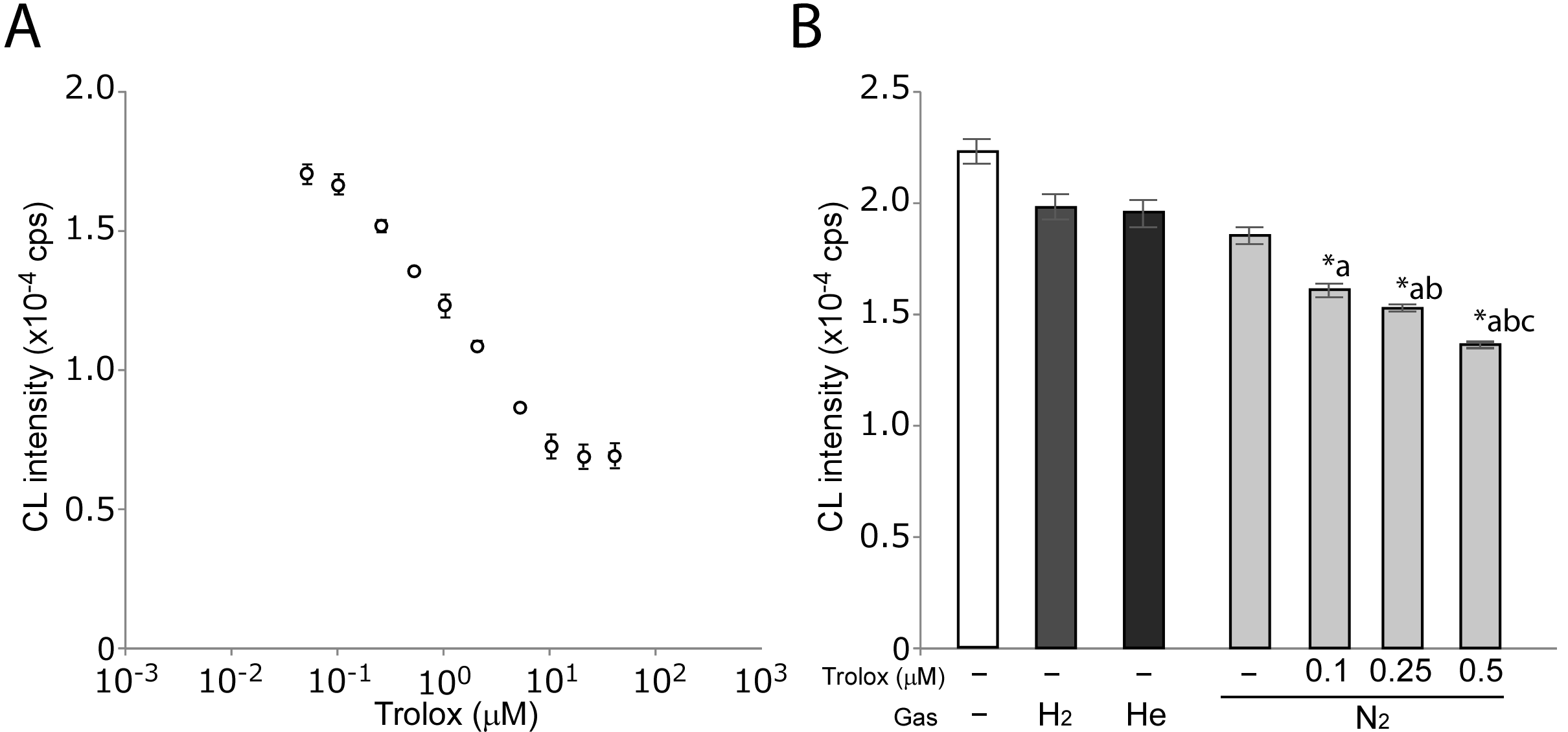

Supplement: S1 Fig — A. Plots of CL intensity versus log concentration of Trolox. We used N2 dissolved water for measurement superoxide anion radical scavenging activity of Trolox. B. Superoxide anion radical scavenging activity of Trolox and sample water. H2, He and N2 represent H2 dissolved water, He dissolved water and N2 dissolved water, respectively. *a, *b and *c indicate p values of < 0.01 when compared with the value for N2 dissolved water, 0.1 μM Trolox-containing and 0.25 μM Trolox-containing N2 dissolved water, respectively. (TIF) [file pone.0171192.s003.tif]

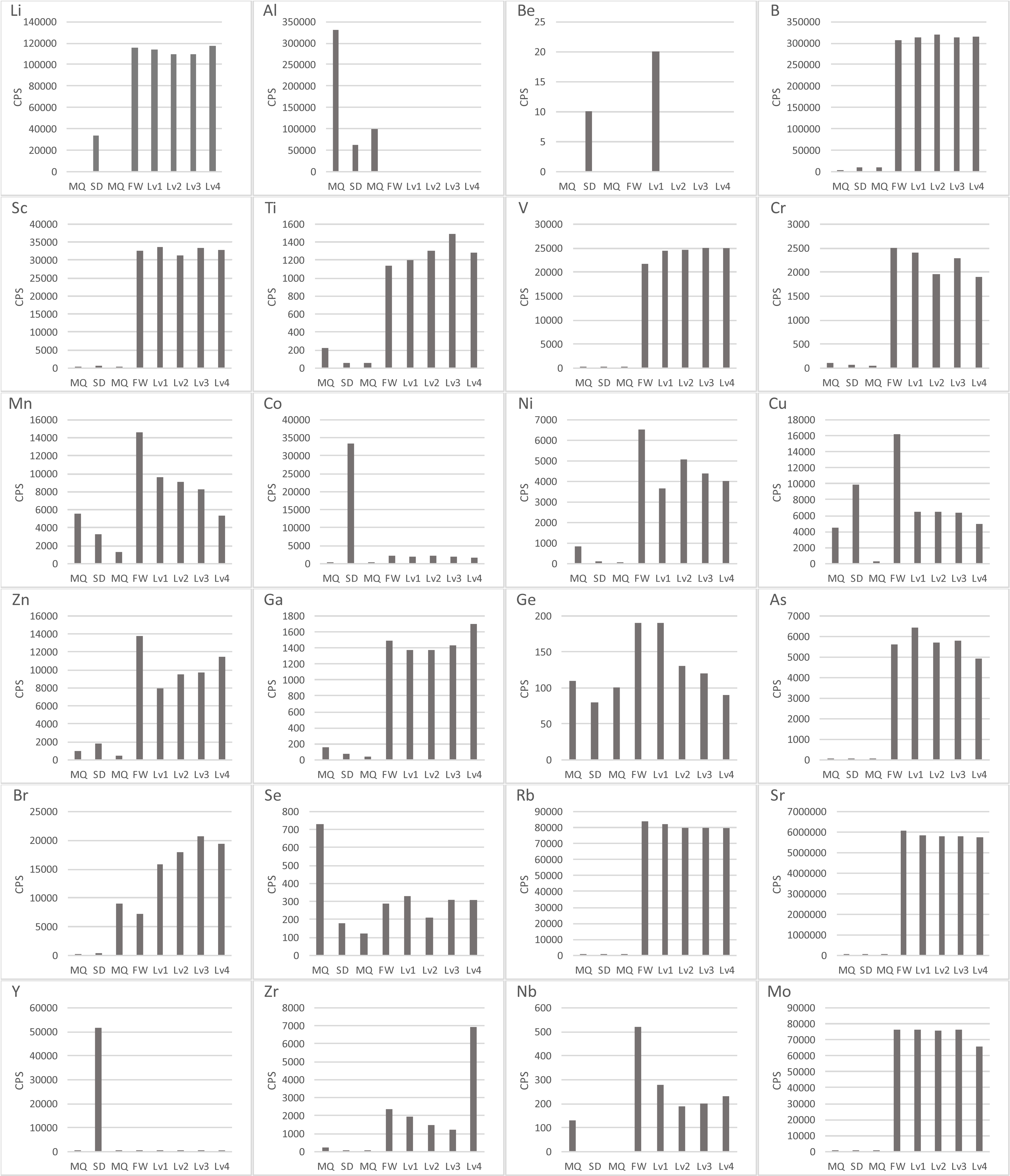

Supplement: S2 Fig — MQ, MilliQ water; FW, filtered water; LV1, LV2, LV3 and LV4, filtered water electrolyzed at levels 1, 2, 3 and 4 with a maximum of 50 V while passing through the gaps between the electrodes; SD, standard solution that contains 10 ppb each of lithium, yttrium and thallium in MilliQ water. (TIF) [file pone.0171192.s004.tif]

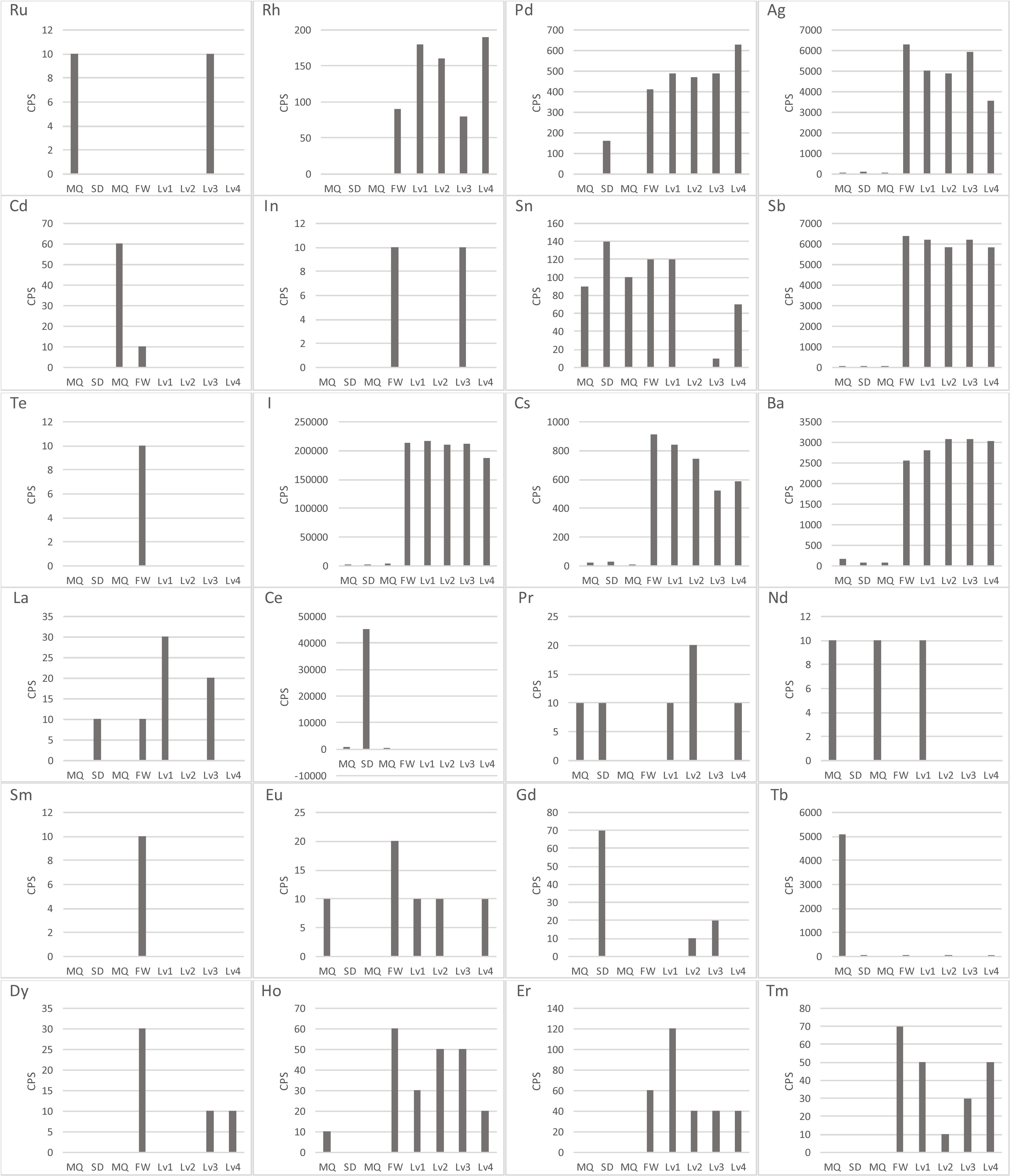

Supplement: S3 Fig — Notations are same as S2 Fig. (TIF) [file pone.0171192.s005.tif]

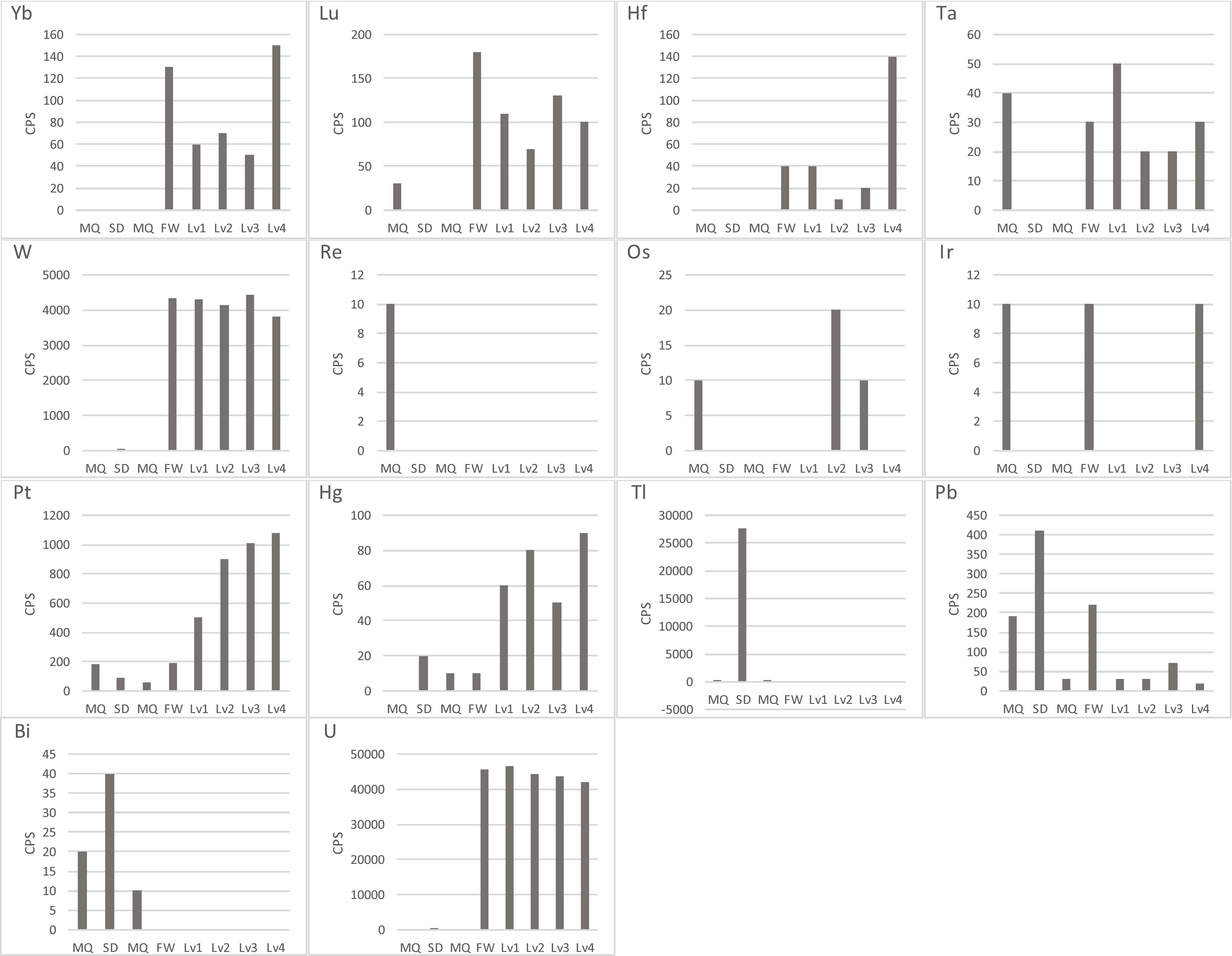

Supplement: S4 Fig — Notations are same as S2 Fig. (TIF) [file pone.0171192.s006.tif]
