# Supplementary material for: Electrochemically reduced water exerts superior reactive oxygen species scavenging activity in HT1080 cells than the equivalent level of hydrogen-dissolved water
Source: PLoS One. 2017 Feb 9;12(2):e0171192. doi: 10.1371/journal.pone.0171192 (PMC5300231; doi:10.1371/journal.pone.0171192)
Supplement: S1 Table — ERWLV1-LV4, ERW generated by electrolyzing filtered water at level 1–4 with a constant electric current of 50 V (upper limit) and a flow rate of 1.0–1.2 l/min. (DOCX) [file pone.0171192.s007.docx]

| **Sample water** | **Treatment** | **Medium pH** |
| --- | --- | --- |
| **MQ** | None | 7.39±0.08 |
| **MQ** | +Mixed gas | 7.41±0.04 |
| **MQ** | +Mixed gas +degas | 7.42±0.03 |
| **FW** | None | 7.45±0.05 |
| **FW** | +Mixed gas | 7.48±0.06 |
| **FW** | +Mixed gas +degas | 7.46±0.02 |
| **FW** | +Mixed gas +autoclave | 7.49±0.05 |
| **ERW_LV1_** | None | 7.49±0.06 |
| **ERW_LV2_** | None | 7.44±0.03 |
| **ERW_LV3_** | None | 7.47±0.07 |
| **ERW_LV4_** | None | 7.47±0.06 |
| **ERW_LV4_** | +degas | 7.42±0.08 |
| **ERW_LV4_** | +autoclave | 7.49±0.04 |
